# The potential application of carbazole-degrading bacteria for dioxin bioremediation

**DOI:** 10.1186/s40643-023-00680-1

**Published:** 2023-08-30

**Authors:** Mai Thi Ngoc Dinh, Van Thi Nguyen, Ly Thi Huong Nguyen

**Affiliations:** 1 Faculty of Biotechnology, Chemistry and Environmental Engineering, Phenikaa University, A9 Building, Nguyen Van Trac Street, Ha Dong District, Hanoi, Vietnam; 2 Bioresource Research Center, Phenikaa University, Hanoi, Vietnam; 3 VNU Institute of Microbiology and Biotechnology, Vietnam National University, E2 Building, 144 Xuan Thuy Street, Cau Giay District, Hanoi, Vietnam; 4 Department of Physiology, College of Korean Medicine, Dongguk University, Gyeongju, Republic of Korea

**Keywords:** Angular dioxygenation, Bacterial degradation, Carbazole, Dioxins

## Abstract

Extensive research has been conducted over the years on the bacterial degradation of dioxins and their related compounds including carbazole, because these chemicals are highly toxic and has been widely distributed in the environment. There is a pressing need to explore and develop more bacterial strains with unique catabolic features to effectively remediate dioxin-polluted sites. Carbazole has a chemical structure similar to dioxins, and the degradation pathways of these two chemicals are highly homologous. Some carbazole-degrading bacterial strains have been demonstrated to have the ability to degrade dioxins, such as *Pseudomonas* sp. strain CA10 và *Sphingomonas* sp. KA1. The introduction of strain KA1 into dioxin-contaminated model soil resulted in the degradation of 96% and 70% of 2-chlorodibenzo-*p*-dioxin (2-CDD) and 2,3-dichlorodibenzo-*p*-dioxin (2,3-DCDD), respectively, after 7-day incubation period. These degradation rates were similar to those achieved with strain CA10, which removed 96% of 2-CDD and 80% of 2,3-DCDD from the same model soil. Therefore, carbazole-degrading bacteria hold significant promise as potential candidates for dioxin bioremediation. This paper overviews the connection between the bacterial degradation of dioxins and carbazole, highlighting the potential for dioxin biodegradation by carbazole-degrading bacterial strains.

## Introduction

Remediation of contaminated sites with chlorinated polyaromatic hydrocarbon and N-heterocyclic aromatic compounds is an urgent problem (Peng et al. 2013). For this purpose, many investigators have attempted the isolation of xenobiotic-degrading bacteria.

Dioxins, which include polychlorinated dibenzo-*p*-dioxins, dibenzofurans (PCDD/Fs) and coplaner polychlorinated biphenyls (PCBs), are highly toxic pollutants that are widespread due to human activities (Hiraishi 2003). They are produced as trace contaminants by various industrial processes and by the burning of solid wastes in municipal incinerators and accidental fires. Exposure to dioxins poses significant risks to human health because they are both toxic and teratogenic (Bertazzi et al. 2001). Because of the toxicity and chemical inertness of dioxins, their removal from polluted environments is one of the most challenging problems in environmental technology (Peng et al. 2013).

Various physicochemical processes, such as photodegradation, thermal remediation, dechlorination with metal catalysts, and dioxin inhibitors such as nitrogen and sulfur compounds in dioxin-contaminated waste have been considered for application of dioxin detoxifcation and degradation (Saibu et al. 2020). Lin and colleagues (2015) utilized thiourea as a dioxin inhibitor in a in a large-scale municipal solid waste incinerator with high capacity (34 t h^−1^) (Lin et al. 2015). Their results show that thiourea reduces the dioxins in flue gas by 55.8 wt.%, those in fly ash by 90.3 wt.% and the total dioxins emission factor by 91.0 wt.%. The highest removal efficiency of PCDD/Fs was 97.24%, achieved using thermal remediation at 300 °C in the study conducted by Liu and co-workers (Liu et al. 2015). Liquid 2,3,7,8-TCDD (tetrachlorodibenzo-p-dioxin) and PCDD/Fs from fly ash were degraded using a process called Ce(III)/Ce(IV) redox couple-mediated electrochemical oxidation. After reacting with Ce(IV), the concentration of the highly toxic liquid isomer 2,3,7,8-TCDD was reduced to 2.96 pg g^−1^ International Toxic Equivalent quantity (I-TEQ) from the initial value of 1200 pg g^−1^ I-TEQ, corresponding to a removal efficiency of 99.75%. The total PCDD/Fs concentrations decreased to 33.16 pg g^−1^ I-TEQ from the initial value of 58 pg g^−1^ I-TEQ, resulting in removal efficiency of 42.8% (Palanisami et al. 2015). Dioxins can also be degraded through the photodegradation technology. In the study by Nguyen et al. 2014, the highest dioxin removal efficiency by photodegradation was 79.6% for the sequential intermittent–continuous UV-exposure experiment with nTiO2 (Nguyen et al. 2014).

Physicochemical processes have proven to be highly effective in the degradation of dioxins. However, these technologies are not economically and ecologically feasible for remedying large areas of polluted soils. For instance, thermal remediation is challenging to apply to non-combustible media such as sediments (Bedard 2008). More often than not, these methodologies result in the introduction of new chemical wastes into the environment (Saibu et al. 2020). As microorganisms play important roles in the degradation and mineralization of xenobiotic and aromatic compounds in natural environments, biological methods using particular microorganisms or microbial consortia capable of dioxin transformation and degradation offer greater potential for environmental remediation compared to physicochemical approaches (Chen et al. 2016; Hiraishi 2003; Maddalwar et al. 2021). Microbial degradation presents a particularly appealing option as it allows for on-site treatment of pollutants without requiring costly and potentially hazardous transportation of contaminated materials. In general, bioprocesses leverage the degradative capabilities of indigenous and/or introduced microorganisms, aiming to ideally mineralize pollutants and yield harmless end products like carbon dioxide, water, and inorganic salts (Halden et al. 2008).

The technique of bioaugmentation, which involves introducing bacteria into a contaminated environment to enhance the degradation of pollutants, has gained significant interest in bioremediation methods. This approach becomes particularly crucial in cases where the indigenous microorganisms are incapable of degrading these xenobiotics, making it the sole viable method for successful bioremediation (Wittich 1998). Consequently, many researchers have focused their studies on the isolation and identification of the bacteria that can degrade xenobiotics, including dioxins.

Several bacterial strains have been identified for their ability to degrade dioxins, as shown in Table 1. However, there are very few bacteria capable of degrading highly substituted dioxin congeners. Therefore, there is need to explore and develop more bacterial strains with unique metabolic potentials which can be applied for effective remediation in dioxin-polluted sites.

**Table 1:** Bacterial strains capable of biodegrading dioxins

Bacterial strains	PCDD/Fs congenes	Concentration	Removal Average (%)	Time	References
*Sphingomonas* sp. HH69	DF	1 gL^−1^	100	40 h	Fortnagel et al. 1990
*Sphingomonas wittichii* RW1	DD	1 mM	81	72 h	Nam et al. 2005
	PCDD	29 ppt	75.5	15 days	
*Nocardioides aromaticivorans*	DF	1.1 mmol mL^−1^	100	48 h	Kubota et al. 2005
*Terrabacter* sp. strain DBF63	2-CDD	10 µg mL^−1^	75	18 h	Habe et al. 2001
	2-CDF		82.5		
*Sphingomonas* sp. XLDN2-5	DF	0.2 mM	100	48 h	Gai et al. 2007
*Pseudomonas putida* B6-2	Biphenyl	15 mmol L^−1^	96	20 h	Li et al. 2009
*Pseudomonas* sp. strain ISTDF1	DF	200 mg L^−1^	85	36 h	Jaiswal et al. 2011
*Rhodococcus* sp strain p52	2-CDF	100 mg L^−1^	70	96 h	Peng et al. 2013
*Agrobacterium* sp. PH-08	DF	2 mmol L^−1^	80	48 h	Le et al. 2014
*Pseudomonas mendocina* strain NSYSU	OCDD (octachorodibezo-*p*-dioxin)	20.1 mg kg^−1^	74	60 days	Lin et al. 2017

The investigation of microbial degradation of dioxins started appearing in 1970s (Kearny et al. 1972; Ward and Matsumura 1978). Over time, significant progress has been made in understanding the mechanism of microbial dioxin degradation, as well as the biodiversity and ecophysiology of microorganisms capable of degrading dioxins (Armengaud and Timmis 1997; Bressler and Fedorak 2000; Nojiri et al. 2001; Nojiri and Omori 2002; Saibu et al. 2020). The existing information suggests that the ring structures of dioxins are degraded by aerobic bacteria that contain aromatic hydrocarbon dioxygenases having a broad substrate specificity (Hiraishi 2003). Interestingly, carbazole-degrading bacteria also possess aromatic ring-cleavage dioxygenases, making them potentially useful for the bioremediation of dioxins. Ring cleavage dioxygenases catalyze the critical ring-opening step in the catabolism of aromatic compounds. The dioxygenation breaks the aromatic ring of dioxins, occurring at the lateral 1,2 positions (occasionally 2,3 or 3,4 positions) or at the angular positions 4 and 4a adjacent to the ether bridge (Hirashi et al. 2003). The dioxygenase of carbazole degrader *Pseudomonas* sp. CA10, for example, catalyze predominantly angular insertion of oxygen. The dioxygenase from another carbazole-degrading strain, *Nocardioides aromaticivorans* IC177, can catalyze both reactions, angular and lateral dioxygenation (Seo et al. 2009).

Carbazole, an environmental pollutant, raises concerns regarding its release into the environment. This nitrogen-containing compound is found in fuels derived from petroleum, oil shale, and tar sand sources (Odabasi et al. 2006). Carbazole is a persistent azarene compound composed of a dibenzopyrrole ring, known for its mutagenic and toxic activity (Jha and Bharti 2002; Jha et al. 2002). Carbazole has a chemical structure similar to dioxins, and the degradation pathways of these two chemicals are highly homologous (Nojiri and Omori 2002). Notably, the enzymes involved in carbazole degradation can also catalyze the respective degradation steps for dioxins (Habe et al. 2001; Nojiri et al. 2003; Sato et al. 1997a). Some studies have explored the degradation of dioxins by carbazole-utilizing bacteria. Therefore, it is of interest to investigate whether carbazole-utilizing bacteria, other than these strains, also have the potential to co-oxidize dioxins.

This article provides an overview of the current knowledge on the microbial degradation of dioxins and carbazole, elucidating their interrelationship. The potential utilization of carbazole-degrading bacteria for the bioremediation of dioxins is discussed.

## General description of dioxins and carbazole

Dioxins are a class of structurally and chemically related polyhalogenated aromatic hydrocarbons that mainly includes polychlorinated dibenzo-*p*-dioxins (PCDDs), dibenzofurans (PCDFs), and co-planar polychlorinated biphenyls (co-planar PCBs). The typical molecular structure of a dioxin consists of two rings of six carbon atoms (benzene rings) bound by oxygen atom(s) with chlorine or hydrogen atoms attached. There are 75 kinds of PCDDs, 135 PCDFs and 209 co-planar PCBs, with variations in molecular shape based on the numbers and locations of the chlorine atoms (Kulkarni et al. 2008). The physical and chemical properties of each dioxin congener differ depending on the degree and position of chlorine substitution. Generally, dioxins are characterized by their chemical stability, low volatility, low solubility in water, long-distance transportability and ability to biomagnify within food chains.

Dioxins, which are halogenated aromatic by-products, primarily originate from various industrial and thermal processes such as waste incineration, bleaching of pulp, metal smelting, synthesis of haloaromatic compounds (Klees et al. 2015; Tue et al. 2016). Additionally, dioxins are also produced naturally by forest fires, volcanic eruptions and biological processes, for example, dumping sites and landfills (Hoekstra et al. 1999). Dioxins have been associated with historical incidences of pollution, such as the case of pollution during the Vietnam War (Hay 1979) and the Seveso industrial accident (Bertazzi et al. 1998). They are widely recognized as highly toxic and carcinogenic compounds with long half-lives, making them among the most problematic environmental pollutants (Hiraishi 2003). Dioxins have been linked to cancer, reproductive and developmental abnormalities, immune system damage, and interference with hormonal systems (Nguyen et al. 2022).

**Fig. 1:**
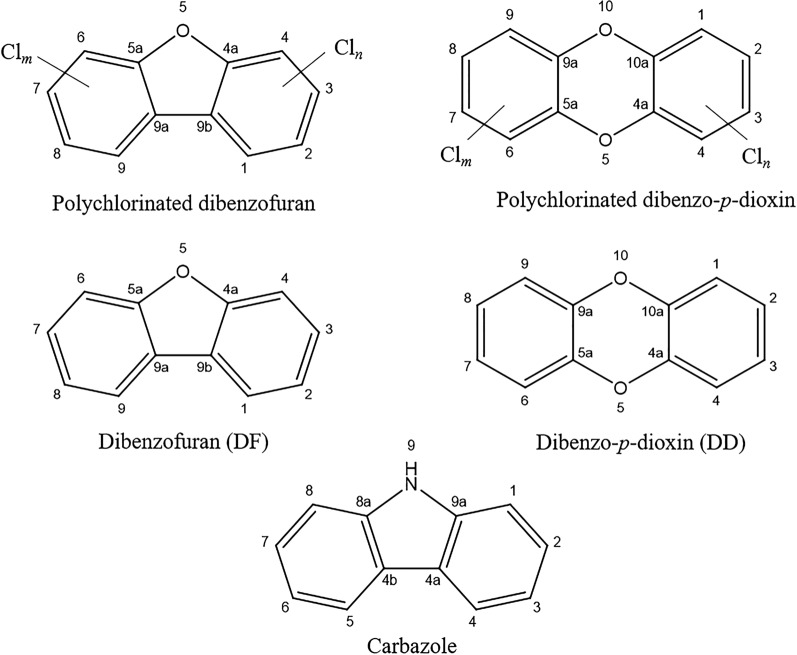
Structure of dioxins and the structurally similar compounds

Carbazole is a structural analog of dioxin (Fig. 1). It is a tricyclic aromatic N-heteroatomic compound, specifically referred to as dibenzopyrrole diphenylenimine, with a chemical formula of C_12_H_9_N and a molecular weight of 167.2. Carbazole exists as a white crystalline solid under normal temperature conditions. It is predominantly found in fossil fuels and can also be found in cigarette smoke, as well as emitted from the combustion of coal and wood (Salam et al. 2017). Despite its significance in the synthesis of several dyes, reagents, insecticides, explosives, lubricants and its application as a color inhibitor in detergents, the environmental dispersion of carbazole poses risks to both the environment and public health due to its mutagenic and carcinogenic effects (Nojiri and Omori 2007; Jha et al. 2002). As illustrated in Fig. 1, carbazole is structurally similar to dioxins, which are by themselves even more toxic and/or mutagenic (Ulbrich and Stahlmann 2004; White and Birnbaum 2009). This means that understanding the mechanisms involved in carbazole biodegradation opens up the possibility of comprehending the biodegradation of dioxins and similar xenobiotics.

## Effective pathway for dioxin and carbazole degradation: angular dioxygenation

Carbazole shares a chemical structure resembling that of dioxins, and the degradation pathways of these compounds exhibit significant homology. Remarkably, enzymes involved in carbazole degradation possess the ability to catalyze the respective degradation steps for dioxins (Habe et al. 2001; Sato et al. 1997a).

**Fig. 2:**
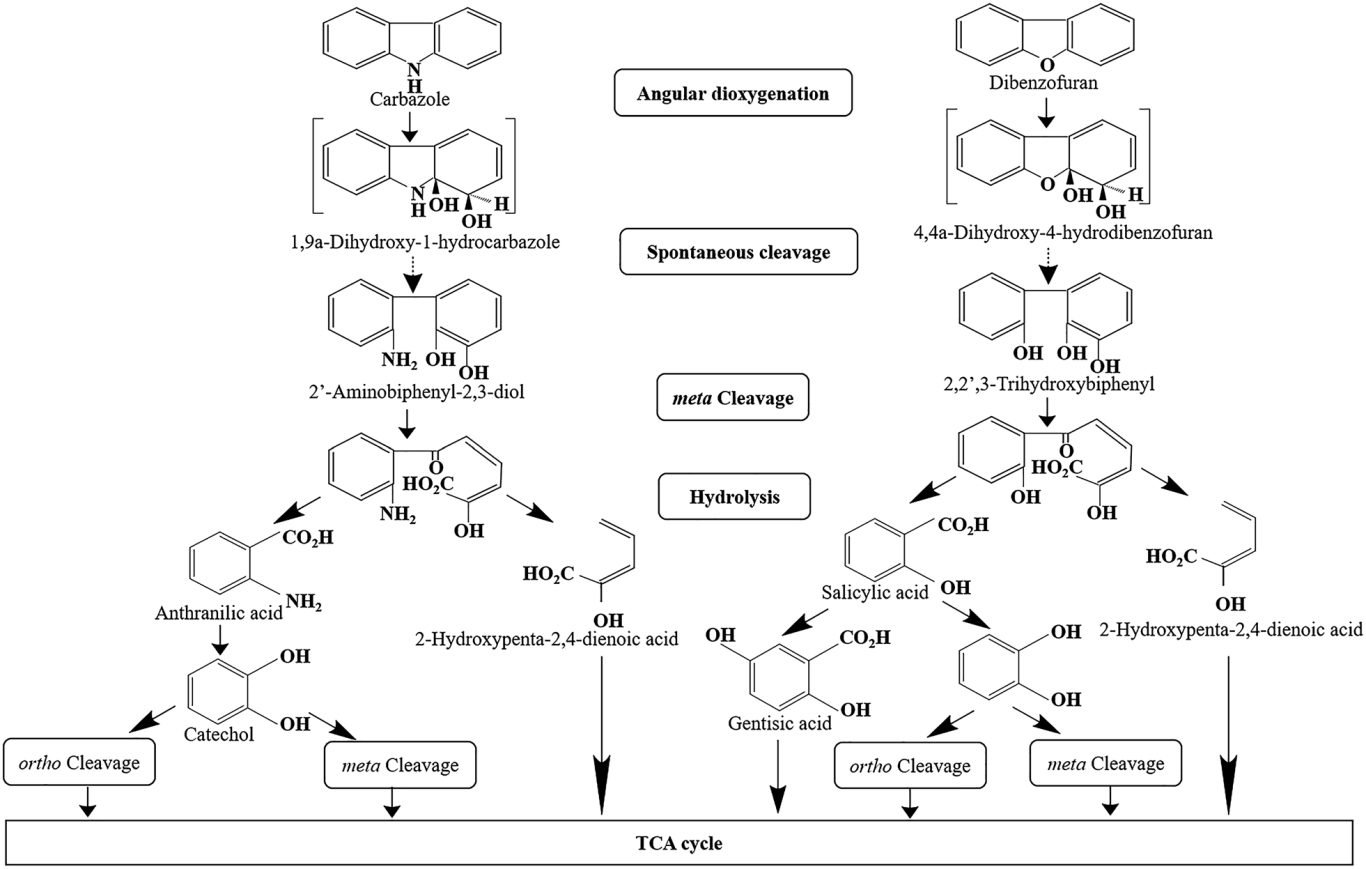
Overview of bacterial degradative pathways for carbazole and dibenzofuran via angular deoxygenation (Nojiri et al. 2001)

Some carbazole utilizing bacteria reported in the literature degrade carbazole via angular dioxygenation. This process leads to the complete mineralization of carbazole, with the resulting catechol being converted into a tricarboxylic acid (TCA) cycle intermediate (Nojiri and Omori 2002). The carbazole degradation pathway for *P. resinovorans* CA10 has been extensively studies. *P. resinovorans* CA10 was isolated from activated sludge of a municipal wastewater treatment facility in Tokyo, Japan (Ouchiyama et al. 1993). The strain is capable of utilizing carbazole as a sole source of carbon, nitrogen and energy. Carbazole undergoes dioxygenation at angular (C9a) and adjacent (C1) carbon atoms, resulting in the formation of an unstable hemiaminal compound known as 1-hydro-1,9a-dihydroxycarbazole. The five-member ring of this compound spontaneously cleaves, generating 2ʹ-aminobiphenyl-2,3-diol. This intermediate is further converted to anthranilate through *meta*-cleavage and subsequent hydrolysis (Fig. 2). Anthranilate undergoes dioxygenation at the C1 and C2 positions, followed by spontaneous deamination and decarboxylation, resulting in the formation of catechol (Kobayashi and Hiyaishi 1970). In *P. resinovorans* CA10, catechol is then converted to a tricarboxylic acid (TCA)-cycle intermediate via an *ortho*-cleavage pathway, although a *meta*-cleavage pathway is known to be an alternative catechol degradation pathway in another carbazole degrader (Ouchiyama et al. 1998).

The metabolic pathway of carbazole is homologous to the degradation route for dioxins, which is initiated by angular dioxygenase attack. The angular pathway knocks down the planar structure of dioxins, ultimately reducing their toxicity (Field and Sierra-Alvarez 2008). *S. Wittichii* strain RW1 is considered to be the best-characterized among organisms with dioxin degradation capability (Nojiri and Omori 2002). This strain was isolated from Elbe River (Germany) through enrichment culture technique. *S. wittichii* RW1 is capable of utilizing both dibenzo-*p*-dioxin and dibenzofuran as the sole source of carbon and energy (Wittich et al. 1992). The angular dioxygenation pathway for dibenzofuran in *S. wittichii* strain RW1 has been proposed. The initial step in the degradation of dibenzofuran and dibenzo-*p*-dioxin is dioxygenation attack at the 4 and 4a positions of one of the aromatic rings, leading to the formation of corresponding *cis*-dihydrodiols. The hemiacetal products derived from dibenzofuran spontaneously transform into 2,2',3-trihydroxybiphenyl (Fig. 2). The dihydroxylated rings of the products are then cleaved at the meta position by an extradiol dioxygenase. Hydrolysis of the *meta*-cleavage products in dibenzofuran metabolic pathway yields salicylic acid (Hiraishi 2003).

The process of angular dioxygenation in dioxins is facilitated by the Rieske non-heme iron oxygenases, referred to as angular dioxygenases. These enzymes act on angular positions (4,4a or 1,10a) adjacent to the ether bridge (Xu et al. 2006). Generally, three dioxygenases have been identified to catalyze the initial reaction of the dioxin catabolic pathway: DF 4,4a-dioxygenase (*dbfA* or *dfdA*) characterized from *Terrabacter* sp. strain DBF63, DD 1,10a-dioxygenase (*dxnA*) obtained from *S. wittichii* RW1 as well as *Rhodococcus* sp. strain HA01 and carbazole 1,9a-dioxygenase (*carAa*) from *P. resinovorans* CA10, all of which attack at the angular position (Field and Sierra-Alvarez 2008). Carbazole 1,9a-dioxygenase (CARDO) of *P. resinovorans* CA10 is a three-component dioxygenase system belonging to the Rieske non-heme iron oxygenase family. CARDO consists of a terminal oxygenase and electron-transport proteins (Nam et al. 2002). The terminal oxygenase component, CARDO-O, is a homotrimeric enzyme that contains one Rieske [2Fe–2S] cluster ([2Fe–2S]_R_) and one active-site iron (Fe^2+^) in a single subunit (*CarAa*) (Nojiri and Omori 2007). The electron-transport proteins of CARDO, which mediate electron transport from NAD(P)H to CARDO-O, consist of ferredoxin (CARDO-F; a monomer of CarAc), which contains one [2Fe–2S]_R_, and ferredoxin reductase (CARDO-R; a monomer of CarAd), which contains one flavin adenine dinucleotide (FAD) and one plant-type [2Fe–2S] cluster ([2Fe–2S]_P_) (Nam et al. 2002; Salam et al. 2017).

CARDO, in addition to its notable angular deoxygenation capability, exhibits diverse oxygenation activities towards aromatic compounds. Biotransformation experiments conducted with *E. coli* cells containing *carAa*, *carAc*, and *carAd* genes have demonstrated that CARDO possesses the ability to catalyze both lateral dioxygenation and monooxygenation reactions on various aromatic substrates, indicating a broad substrate specificity (Salam et al. 2017; Takagi et al. 2002). Through the analysis of oxygenation products obtained from these biotransformation experiment, it has been observed that carbazole 1,9a-dioxygenase is capable of catalyzing the angular dioxygenation reactions on carbazole, dibenzofuran, dibenzo-*p*-dioxin, xanthene, and phenoxathiin. However, it does not exhibit angular dioxygenation activity towards 9-fluorenone or dibenzothiophene. This suggests that angular dioxygenation occurs preferably at positions adjacent to oxygen or nitrogen atoms rather than those adjacent to a sulfur or carbon atom (Nojiri and Omori 2007; Nojiri 2012).

## Diversity of carbazole-degrading bacteria having the *car* gene cluster

Over the years, numerous strains capable of degrading carbazole have been isolated, with *P. resinovorans* CA10 being one the most extensively studied since its isolation from municipal waste water. Genetic analysis of carbazole-degrading bacteria has revealed that many strains belonging to the genera *Pseudomonas*, *Burkholderia*, and *Janthinobacterium* possess nearly identical carbazole degradation genes with *car* gene cluster from *P. resinovorans* CA10 (Nojiri and Omori 2007). Although these carbazole degraders originate from different sources, a comparison of the gene organization and flanking regions of their *car* gene clusters indicates evolutionary diversity as reflected in differences in copy number of *car* gene cluster among carbazole degraders (Inoue et al. 2004). This diversity may arise due to the presence of *car* gene clusters on plasmids or transposons, as well as their flanking regions containing IS (insertion sequence) elements (Inoue et al. 2004).

Although there is relatively low homology (< 60% nucleotide sequence identity) between the *car* gene cluster counterparts, gene cluster similar in organization and phylogeny to the *car* gene cluster from *P. resinovorans* CA10 have been identified in the genera *Sphingomonas* and *Nocardioides*. Unlike the *car* gene cluster from *P. resinovorans* CA10, *car* gene clusters from *Sphingomonas* sp. GTIN11 and *Sphingomonas* sp. KA1 (reclassified as *Novosphingobium* sp. KA1) lack the NAD(P)H:ferredoxin oxidoreductase gene involved in the initial dioxygenase, but contain the genes for terminal oxygenase (*carAa*) and ferredoxin (*carAc*), *meta*-cleavage enzyme (*carBaBb*), and HOADA hydrolase (*carC*). While *Sphingomonas* CarAa shares significant homology (> 55% identity) with CA10 CarAa, ferredoxin (CarAc) in *Sphingomonas* does not exhibit similarity to the Rieske ferredoxin, including CarAc_CA10_, but shows resemblance to the putidaredoxin-type ferredoxins. Notably, CarAa_KA1_ can receive electrons from CarAc_KA1_ and catalyze the angular dioxygenation of carbazole, indicating a difference in ferredoxin selectivity between CarAa_CA10_ and CarAa_KA1/GTIN11_. The *car*_KA1_ gene cluster (re-designated *car*-I_KA1_) was found on plasmid pCAR3, and recent research has revealed the presence of an additional copy of the *car* gene cluster (*car*-II_KA1_ gene cluster) (Habe et al. 2002; Urata et al. 2006). Additionally, NAD (P)H:ferredoxin oxidoreductase genes (*fdr*I and *fdr*II), and a third putidaredoxin-type ferredoxin gene were identified on pCAR3 (Urata et al. 2006). Further investigation by Inoue and co-workers has shown that homologues of the *car*_KA1/GTIN11_ gene cluster exist in various *Sphingomonas* and related strains (Table 2) (Inoue et al. 2004, 2005).

**Table 2:** Diversity of carbazole-degrading bacteria having the *car* gene cluster

Bacterial strains	Products	Genetic analysis	References
*P. resinovorans* CA10	Anthranilate, catechol	*car* (P), pCAR1	Ashikawa et al. 2005; Sato et al. 1997a
*P. resinovorans* CA06	Anthranilate, catechol	*car* (P)	Ouchiyama et al. 1993
*Pseudomonas* sp. LD2	Anthranilate	*car* (P)	Gibbs et al. 2003
*Sphingomonas* sp. CB3	Not detected	*car* (SC)	Shepherd and Lloyd-Jones 1998
*P. stutzeri* OM1	Anthranilate	*car* (P)	Shintani et al. 2003
*Sphingomonas* sp. GTIN11	Anthranilate	*car* (SK)	Kilbane et al. 2002
*Sphingomonas* sp. KA1	None	*car* (SK), pCAR3	Habe et al. 2002; Shintani et al. 2007
*Janthinobacterium* sp. J3	None	*car* (P)	Inoue et al. 2004
*Janthinobacterium* sp. J4	None	*car* (P)	Inoue et al. 2004
*Pantoea* sp. J14	None	*car* (P)	Inoue et al. 2004
*Novosphingobium* sp. J30	None	*car* (P)	Inoue et al. 2004
*Pseudomonas* sp. J11	None	*car* (P)	Inoue et al. 2004
*Pseudomonas* sp. K15	None	*car* (P)	Inoue et al. 2004
*Pseudomonas* sp. K22	None	*car* (P)	Inoue et al. 2004
*Pseudomonas* sp. K23	None	*car* (P)	Inoue et al. 2004
*Sphingomonas* sp. M2	None	*car* (SK)	Inoue et al. 2004
*P. putida* HS01	None	*car* (P), pCAR2	Shintani et al. 2005
*Pseudomonas* sp. IC017	None	*car* (P)	Inoue et al. 2005
*Pseudomonas* sp. IC033	None	*car* (SK), *car* (SC)	Inoue et al. 2005
*Burkholderia* sp. IC049	None	*car* (P)	Inoue et al. 2005
*Achromobacter* sp. IC074	None	*car* (SK)	Inoue et al. 2005
*Sphingomonas* sp. IC075	None	*car* (SK)	Inoue et al. 2005
*Sphingomonas* sp. IC081	None	*car* (SK), *car* (SC)	Inoue et al. 2005
*Sphingomonas* sp. IC097	None	*car* (SK)	Inoue et al. 2005
*Sphingomonas* sp. IC145	None	*car* (SK)	Inoue et al. 2005
*Janthinobacterium* sp. IC161	None	*car* (P)	Inoue et al. 2005
*N. aromaticivorans* IC177	Anthranilate	*car* (N)	Inoue et al. 2006
*Stenotrophomonas* sp. IC193	None	*car* (SK)	Inoue et al. 2005
*Sphingomonas* sp. IC209	None	*car* (SK)	Inoue et al. 2005
*Sphingomonas* sp. IC258	None	*car* (SK)	Inoue et al. 2005
*Sphingomonas* sp. IC268	None	*car* (SK)	Inoue et al. 2005
*Sphingomonas* sp. IC273	None	*car* (SK)	Inoue et al. 2005
*Sphingomonas* sp. IC290	None	*car* (SK)	Inoue et al. 2005
*Sphingomonas* sp. IC291	None	*car* (SK)	Inoue et al. 2005
*Sphingomonas* sp. IC300	None	*car* (SK)	Inoue et al. 2005
*Sphingomonas* sp. IC306	None	*car* (SK)	Inoue et al. 2005
*Sphingomonas* sp. IC315	None	*car* (SK)	Inoue et al. 2005
*Sphingomonas* sp. IC321	None	*car* (SK)	Inoue et al. 2005
*Neptuniibacter sp. strain* CAR-SF	None	*car* (P)	Nagashima et al. 2010
*Lysobacter sp. OC7*	None	*car* (OC7)	Maeda et al. 2009
*Lysobacter sp. OC9*	None	*car* (OC9)	Maeda et al. 2010b
*Erythrobacter* sp. KY5	None	*Caulobacteridae*-type I *car*	Vejarano et al. 2018
*Thalassococcus* sp. S3	None	*Caulobacteridae*-type II *car*	Vejarano et al. 2019

Inoue and co-workers documented the isolation of the first Gram-positive carbazole degrader, *N. aromaticivorans* IC177, from soil (Inoue et al. 2005). Their study revealed the presence of a complete *car* gene cluster in this strain, which was organized as *carRcarAaCBaBbAcAd*. Additionally, the *meta*-pathway genes *carDFE* were found to be closely linked and located upstream the main *car* gene cluster. The *car* genes in strain IC177 (*car*_IC177_) exhibited a more streamlined or “optimized” structure compared to those from the Gram-negative CA10 and KA1 strains, as evidenced by the overlapping of stop and start codons of neighboring genes. The CARDO_IC177_ strain belonged to class IIB, characterized by the presence of a Rieske-type ferredoxin and a GR-type reductase. At the time, it was hypothesized that anthranilate degradation of strain IC177 also occurred through dioxygenation mediated by homologs of previously reported anthranilate dioxygenase, although these genes were not detected. It was also reported that, in comparison to Gram-negative degraders, CARDO in strain IC177 displayed a stronger preference for carbazole over other substrates such as biphenyl and dibenzofuran (Inoue et al. 2006).

*Sphingomonas* sp. CB3 has been found to possess a distinct carbazole-degradative *car* gene cluster (Sauber et al. 1977). The gene organization and phylogeny of *car*_CB3_ are not similar to other *car* gene clusters, but it shows a significant similarity to the biphenyl degradative *bph* gene cluster (Shepherd and Lloyd-Jones 1998). Although the carbazole metabolic activity of the enzymes encoded in the *car*_CB3_ gene cluster has not been confirmed, its transcription was detected when carbazole was used as a sole carbon source by CB3 (Nojiri and Omori 2007). Recently, it was reported that *Sphingomonas* carbazole degraders of strains IC033 and IC081 had both homologues of *car*-I_KA1_ and *car*_CB3_ (Table 2) (Inoue et al. 2005). Additionally, Southern hybridization analysis demonstrated the presence of a *car*-I_KA1_ homologue in the genome of CB3 as well (Inoue et al. 2005).

Other carbazole degraders described to date include *S. yanoikuyae* strain XLDN2-5, which is not only capable of degrading carbazole, but also can degrade dibenzofuran and dibenzothiophene when carbazole is present as an inducer. This strain has a *car* gene cluster structure similar to that of strain KA1, as well as similar genes involved anthranilate degradation, although genes for the *meta*-cleavage of catechol were also found. (Gai et al. 2007; 2010; 2011). Another carbazole degrader, *P. stutzeri* strain OM1, exhibits an identical *car* gene cluster structure to that of strain CA10, but with the notable difference that it is located on its chromosome (Ouchiyama et al. 1998; Shintani et al. 2003).

**Fig. 3:**
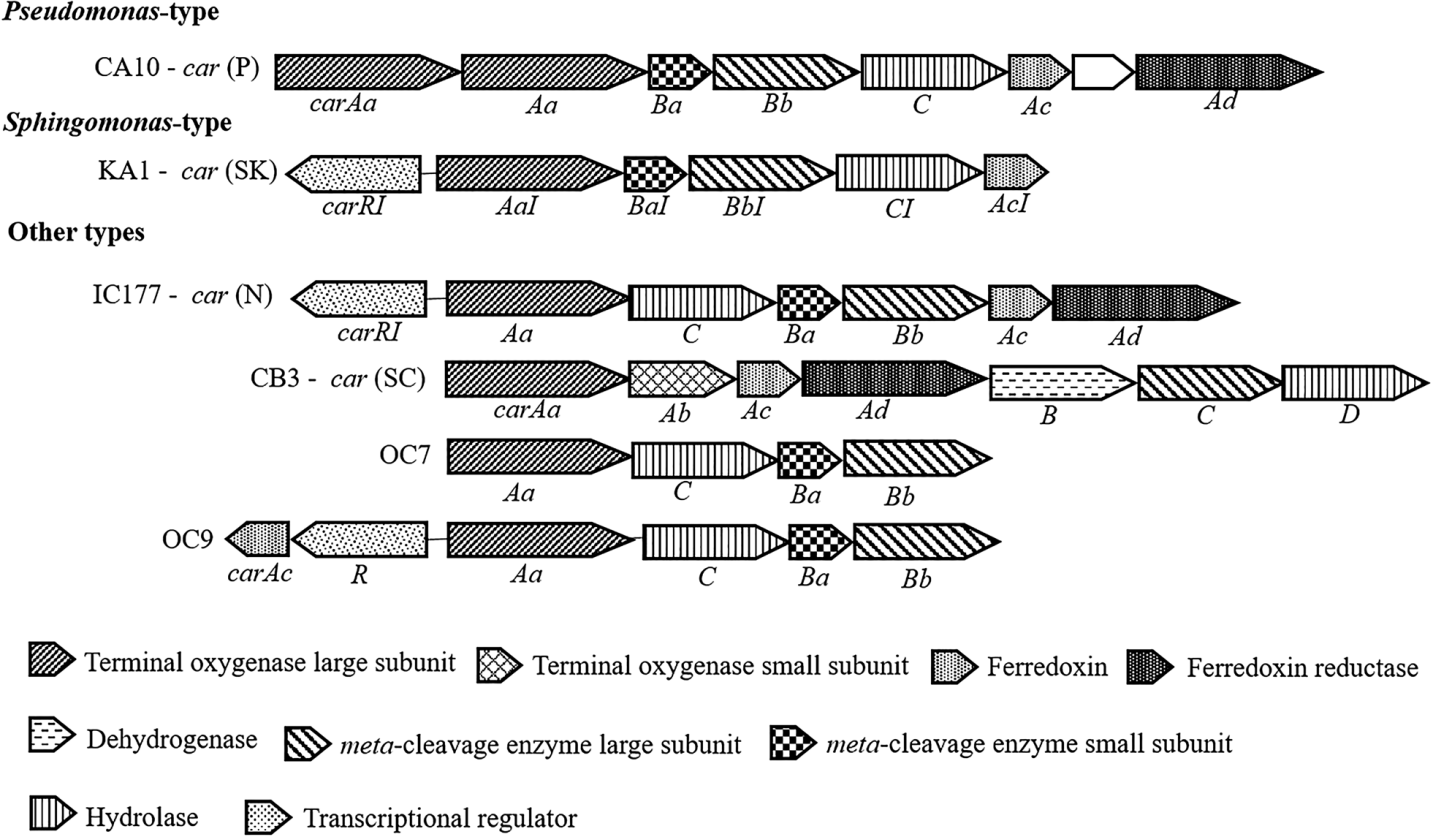
Types of *car* gene clusters as found in different strains (Maeda et al. 2010b). The lower part shows the meaning of the fill patterns from each ORF indicated by the pentagons

While most studies have focused on bacteria isolated from onshore sites, some have aimed to explore novel components in the *car* gene cluster from marine isolates. Analysis of the *car* gene cluster in marine carbazole degraders belonging to different genera, including *Neptuniibacter*, *Erythrobacter*, *Terrabacter*, *Lysobacter*, revealed that they lack *car* genes highly similar to *car*_CA10_ and *car*_KA1_ (Salam et al. 2017). The *car* gene cluster in *Neptuniibacter* sp. strain CAR-SF is organized as *carAaBaBbC*, resembling the arrangement observed in *Pseudomonas* and *Sphingomonas*-type car gene clusters, with a 48–77% similarity to the *car*_CA10_ gene (Nagashima et al. 2010). However, in comparison to the *car*_CA10_ gene cluster, the *car*_CAR-SF_ gene cluster lacks the ferredoxin *carAc* and ferredoxin reductase *carAd* genes (Nagashima et al. 2010). *Lysobacter* sp. OC7 is capable of utilizing carbazole, phenanthrene and naphthalene as sole carbon sources. The *car* gene cluster in *Lysobacter* sp. OC7 is arranged as *carAaCBaBb*, with the position of *carC* and *carBaBb* inverted when compared to their positions in *Pseudomonas* and *Sphingomonas*-type *car* gene clusters. However, the gene arrangement follows the same order as in the *car* gene cluster of strain IC177. The open reading frames (ORFs) containing the *car* gene cluster of strain OC7 share 39–52% similarity with *carAa*, *carC*, *carBa*, and *carBb* genes of strains CA10 and KA1, but no similarity with *car* genes of strain CB3, indicating that the *car* genes of strain OC7 are phylogenetically distinct from previously reported *car* gene products (Maeda et al. 2009). In a subsequent study, *Kordiimonas* sp. OC9 was isolated and it was found that its CarAc contains a chloroplast-type [2Fe-2S] center, which is similar to the [2Fe-2S]_4cys_ but with a characteristic difference in the number of amino acid residues separating the iron-coordinating cysteine residue pairs. This represents the first report of a chloroplast-type ferredoxin component in a CARDO system (Maeda et al. 2010a; Ito et al. 2011). Upon cloning the degradative genes into constructed expression plasmids, it was discovered that CARDO_OC9_ exhibited activity towards naphthalene, phenanthrene, biphenyl, fluorene, dibenzofuran and dibenzo-*p*-dioxin (Maeda et al. 2010a). *Janibacter* sp. OC11 was later identified as the first marine Gram-positive carbazole degrader with a CARDO belonging to the class IIB like the one in strain IC177 (Oba et al. 2014). Based on their diversity, known *car* gene clusters identified through heterologous expression and southern hybridization experiments were classified into three types: *Pseudomonas*-type (*car* (P)), which shares homology with the *car* gene in *P. resinovorans* CA10; *Sphingomonas*-type *car* (SK), which shares homology with the *car* gene in *Sphingomonas* sp. KA1; and other types including *car* (N) found in *N. aromaticivorans* IC177, *car* (SC) with homology to *Sphingomonas* sp. CB3, *car* (OC7) found in *Lysobacter* sp. OC7, and *car* (OC9) present in *Kordiimonas* sp. OC9 (Fig. 3). This classification provided a convenient way to visualize the diversity of car gene clusters discovered up until that time (Maeda et al. 2010b).

Recently, the genomes of newly isolated carbazole-degrading strains were sequenced using Illumina’s PCR-free and long insert mate-pair DNA libraries in the MiSeq sequencer, followed by manual in silico contig gap-closing. Two new types of *car* gene cluster were found in surface seawater bacteria: *Caulobacteridae*-type I (Vejarano et al. 2018) and *Caulobacteridae*-type II *car* (Vejarano et al. 2019) gene clusters. Unlike previously described carbazole degraders, *Erythrobacter* sp. KY5 (*Caulobacteridae*-type I *car* gene cluster) lack a CARDO ferredoxin gene carAc either inside the *car* gene cluster or in its vicinity (Vejarano et al. 2018). This could indicate that this strain utilize a novel two-component CARDO system that only requires CarAa and CarAd, or it might suggest that, unlike other strains with a functional RO, the gene for the ferredoxin component is located at a distant locus from that of the terminal oxygenase. On the other hand, *Thalassococcus* sp. S3 (*Caulobacteridae*-type II *car* gene cluster) harbors a putative CARDO ferredoxin reductase gene carAd in the vicinity of the *car* gene cluster (Vejarano et al. 2019). However, unlike other RO reductases, this gene has an additional iron sulfur dicluster of 4Fe–4S configuration. Moreover, the *Caulobacteridae*-type *car* gene clusters are associated with a previously uncharacterized gene cluster, known as the *aca* gene cluster, in carbazole degraders, which is involved in the degradation of anthranilate through a coenzyme A intermediate (anthraniloylCoA). This *aca* gene cluster was also found in Gram-positive bacteria of the genus *Nocardioides* (Vejarano et al. 2019).

## Carbazole degraders as candidate bacteria for dioxin bioremediation

### Pseudomonas sp. strain CA10

**Fig. 4:**
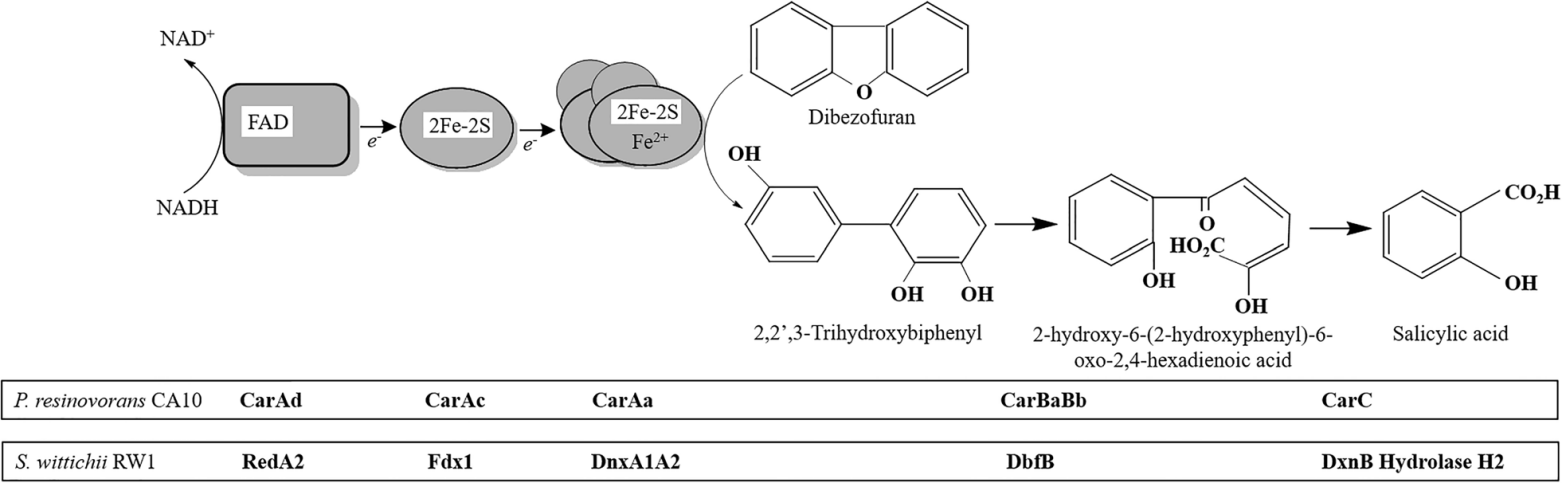
Dibenzofuran degradation by the enzyme systems harbored by *Sphingomonas wittichii* RW1 or *Pseudomonas resinovorans* CA10 (Nojiri and Omori 2002)

In 1993, *P. resinovorans* CA10, a bacterium capable of utilizing carbazole as sole source of carbon, nitrogen, and energy, was isolated from activated sludge of a municipal waste water treatment facility in Tokyo (Ouchiyama et al. 1993). Using shotgun cloning with *meta*-cleavage activity, Sato and colleagues successfully cloned the genes responsible for carbazole conversion to anthranilate from the *P. resinovorans* CA10 (Sato et al. 1997a). The resulting gene fragment contained seven degradative genes, one open reading frame (ORF) encoding a putative protein or unknown function, and two partial possible genes. Functional analysis of these degradative genes revealed the presence of two identical copies each of *carAa*, *carAc*, and *carAd*, which encode terminal oxygenase, ferredoxin, and ferredoxin reductase components of carbazole 1,9a-dioxygenase (CARDO); *carBa* and *carBb*, which encode the structural and catalytic subunits of the *meta*-cleavage enzyme (2’- aminobiphenyl-2,3-diol 1,2-dioxygenase); and *carC*, which encodes the *meta*-cleavage compound (HOADA) hydrolase (Sato et al. 1997a; 1997b). These three enzymes in carbazole degradation have been found to have analogous functions in the degradation of dioxins as the counterparts of the corresponding enzymes in dibenzofuran (dibenzo-*p*-dioxin)-degrading bacteria (Habe et al. 2001; Nojiri et al. 1999). The functions of the carbazole enzymes of strain CA10 in dibenzofuran degradation may be as shown in Fig. 4.

The *car* gene clusters were identified on the 199-kb circular plasmid called pCAR1 (Nojiri et al. 2001). Since catechol, formed from carbazole, is metabolized through the *ortho* cleavage pathway encoded by the chromosomal *cat* operon, it is believed that strain CA10 acquired a metabolic capacity for dioxins and carbazole by recruitment of pCAR1 (Nojiri et al. 2002). The pCAR1-like plasmids containing homologous *car* gene cluster have been detected in other bacteria capable of degrading both dioxins and carbazole, although chromosomally encoded *car* gene clusters were found as well. This phenomenon suggests that pCAR1 plays a significant role in the distribution of dioxins and carbazole degradation capabilities in the environment. The complete nucleotide sequence of pCAR1 (199,035 bp) was analyzed (Nojiri and Omori 2002). The presence of the homologous genes to the *trh* and *tra* genes, which are involved in the conjugal transfer of the plasmids Rts1 from *Proteus vulgaris* and R27 from *Salmonella strains,* suggests that pCAR1 may be a self-transmissible plasmid (Murata et al. 2002; Sherburne et al. 2000).

Interestingly, it was also shown that the entire *car* locus is contained in a 73-kb-long transposon, Tn*4676*. The transposase and cointegrate resolution proteins show close similarity to those found in the toluene- and xylene-degrading transposon, Tn*4651*, which is present in the TOL plasmid pWW0. However, the replication and maintenance systems of pCAR1 are unrelated to those of pWW0 (Nojiri and Omori 2002). Because several bacterial strains have Tn*4676* inserted on their chromosome, both the plasmid pCAR1 and Tn*4676* play significant roles in the distribution of the degrading capacity for dioxins and carbazole in the environment (Nojiri and Omori 2002).

Habe and colleagues successfully carried out bioremediation of actual dioxin-contaminated soil that was contaminated mainly by tetra- to octachlorinated dioxins, by using the soil slurry system and *P. resinovorans* strain CA10 cells (Habe et al. 2001). The soil slurry, prepared by mixing dioxin-contaminated soil from an incinerator site with water at a ratio of 1:5 (wt/vol), was incubated with cells of strain CA10 that were grown on carbazole. Over a 7-day incubation period, the total amount of chlorinated dibenzofuran and dibenzo-*p*-dioxin congeners and toxicity equivalency quantity (TEQ) decreased from 725 to 665 ng/g soil and 11 to 9.4 ng TEQ/g soil, respectively, through a single inoculation of strain CA10 cells at a concentration of 10^11^ CFU/g dry soil. Although the degradation rate of total dioxins was 8.3%, strain CA10 exhibited the ability to transform tetra- to heptachlorinated congeners, including the most toxic compound 2,3,7,8-tetrachlorinated dibenzo-*p*-dioxin.

### Sphingomonas sp. KA1

*Sphingomonas* sp. (reclassified as *Novosphingobium* sp.) KA1 was obtained from the lagoon sludge treated with soft drink wastewater in Chiba Prefecture, Japan (Habe et al. 2002). This strain is capable of utilizing carbazole as a sole carbon, nitrogen, and energy source. *Sphingomonas* sp. strain KA1 does not possess the gene encoding the terminal oxygenase component (*carAa*) of carbazole 1,9a-dioxygenase at high homology (more than 90% identity) to that of *P. resinovorans* strain CA10. However, PCR experiments using the primers for amplifying the internal fragment of the *carAa* gene (810 bp for strain CA10) resulted in the amplification of a PCR product with an unexpected size of 1100 bp. Further sequence analysis revealed that this DNA region contained the portion of two possible ORFs showing moderate homology to *CarAa* and *CarBa* from strain CA10, with amino acid identities of 61% and 40%, respectively (Habe et al. 2002).

The carbazole-degradative (*car*) genes of strain KA1 are located on the large plasmid pCAR3 (Habe et al. 2002; Urata et al. 2006). The complete 254,797-bp nucleotide sequence of the plasmid pCAR3 has been determined for this strain (Shintani et al. 2007). A specific region of approximately 65 kb involved in replication and conjugative transfer shares similarity with a region found in plasmid pNL1 from the aromatic-degrading *N. aromaticivorans* strain F199 (Romine et al. 1999). The presence of numerous insertion sequences, transposons, repeat sequences, and their remnants suggests a high flexibility of this plasmid in genetic structure. Several degradative genes are present on pCAR3, including two types of carbazole-degradative gene clusters (*car*-I and *car*-II), as well as genes encoding electron transfer components of initial carbazole oxygenase (*fdxI*, *fdrI*, and *fdrII*). Putative genes involved in the degradation of anthranilate (*and*), catechol (*cat*), 2-hydroxypenta-2,4-dienoate (*carDFE*), dibenzofuran/fluorene (*dbf/fln*), protocatechuate (*lig*), and phthalate (*oph*) were also identified. It appears that pCAR3 may harbor clustered genes (*car*-I, *car*-II, *fdx*I, *fdr*I, *fdr*II, *and*, and *cat*) responsible for the conversion of carbazole into tricarboxylic acid cycle intermediates. Reverse transcription-PCR analysis further revealed that the transcription of *car*-I, *car*-II, and *cat* genes was induced by carbazole or its metabolic intermediate. Southern hybridization analyses with probes prepared from *car*-I, *car*-II, *rep*A, *par*A, *tra*I, and *tra*D genes indicated that several *Sphingomonas* carbazole degraders possess DNA regions similar to parts of pCAR3 (Shintani et al. 2007).

Habe and colleagues conducted an investigation into the suitability of strain KA1 for the remediation of dioxin-contaminated soil. The carbazole-grown strain KA1 was inoculated into a model soil contaminated with 2-CDD at a density of 10^9^ CFU/g soil and incubated at 30℃ for various periods (Habe et al. 2002). Although 2-CDD levels was decreased in both the presence and absence of strain KA1, the degradation of 2-CDD was enhanced by the addition of strain KA1. Particularly within one day, strain KA1 inoculation resulted in the degradation of 85% of 2-CDD, compared to 46% in the control. Conversely, a different tendency was observed in a model soil contaminated with 2,3-DCDD. In the soil slurry incubated with strain KA1 at a density of 10^9^ CFU/g soil, 2,3-DCDD exhibited gradual degradation, with 70% degradation observed by the 7th day. Generally, the introduction of strain KA1 into dioxin-contaminated model soil resulted in the degradation of 96% and 70% of 2-CDD and 2,3-DCDD, respectively, after 7-day incubation period. These degradation rates were comparable to those obtained using strain CA10 (96% of 2-CDD and 80% of 2,3-DCDD were removed from the same model soil), despite the different carbazole 1,9a-dioxygenase gene present in each strain (Habe et al. 2002).

## Conclusion

Dioxins, highly toxic and carcinogenic compounds with extended half-lives, pose significant environmental pollution challenges. It is crucial to discover and develop bacterial strains with unique metabolic capabilities for effective dioxin bioremediation. In addition to isolating new strains from polluted unexplored environments, the utilization of strains with homologous degradation pathways holds considerable promise. Carbazole-degrading bacteria are particularly promising candidates for dioxin bioremediation. The carbazole degraders, *Pseudomonas* sp. strain CA10 and *Sphingomonas* sp. KA1, have been demonstrated to have the capability of remediating dioxin-contaminated soil. The elucidation of dioxin biodegradation pathways in several carbazole degraders has provided valuable insights. Molecular tools have been instrumental in identifying genes involved in the dioxin metabolic pathway of carbazole utilizing bacteria. Advances in biomolecular engineering have expedited the natural evolutionary process to degrade xenobiotic pollutants. The soil slurry and microcosm laboratory experiments, followed by field studies are clearly necessary to exploit bioremediation techniques for practical applications.

## Data Availability

All data generated or analyzed during this study are included in this published article.
